# SiX_2_ (X = S, Se) Single Chains and (Si–Ge)X_2_ Quaternary Alloys

**DOI:** 10.1021/acsnano.4c04184

**Published:** 2024-06-26

**Authors:** Yangjin Lee, Young Woo Choi, Linxuan Li, Wu Zhou, Marvin L. Cohen, Kwanpyo Kim, Alex Zettl

**Affiliations:** † Department of Physics, University of California at Berkeley, Berkeley, California 94720, United States; ‡ Materials Sciences Division, Lawrence Berkeley National Laboratory, Berkeley, California 94720, United States; § Department of Physics, Yonsei University, Seoul 03722, Korea; ∥ Center for Nanomedicine, Institute for Basic Science, Seoul 03722, Korea; ⊥ School of Physical Sciences and CAS Key Laboratory of Vacuum Physics, University of Chinese Academy of Sciences, Beijing 100049, People’s Republic of China; # Kavli Energy NanoSciences Institute at the University of California at Berkeley, Berkeley, California 94720, United States

**Keywords:** one-dimensional materials, silicon dichalcogenides, nanotubes, atomic chain, transmission electron microscopy, density functional theory

## Abstract

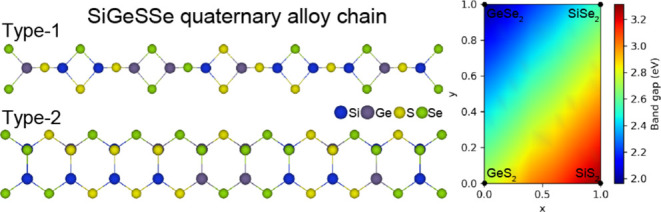

Layered or chain materials have received significant research attention owing to their interesting physical properties, which can dramatically change when the material is thinned from bulk (three-dimensional) to thin two-dimensional sheet or one-dimensional (1D) chain form. Materials with the stoichiometry AX_2_ with A = Si or Ge and X = S or Se form an especially intriguing semiconducting class. For example, bulk silicon dichalcogenides (SiX_2_) consist of 1D chains held together by van der Waals forces. Although this structural configuration has the potential to reveal interesting physical phenomena within the 1D limit, obtaining SiX_2_ single chains has been challenging. We here examine experimentally and theoretically SiX_2_ materials in the low chain number limit. Carbon nanotubes serve as growth templates and stabilize and protect the structures, and atomic-resolution scanning transmission electron microscopy directly identifies the atomic structure. Two distinct chain structures are observed for SiX_2_. Si_*x*_Ge_1–*x*_S_2(1–*y*)_Se_2*y*_ quaternary alloy chains are also synthesized and characterized, demonstrating tunable semiconducting properties at the atomic-chain level. Density functional theory calculations reveal that the band gap of these alloy chains can be widely tuned through composition engineering. This work offers the possibilities for synthesizing and controlling semiconductor compositions at the single-chain limit to tailor material properties.

## Introduction

The investigation of reduced dimensionality materials has aroused intensive experimental and theoretical interest owing to their extraordinary physical characteristics, such as optical, electrical, thermal, and magnetic properties, which differ significantly from those of bulk forms.^1−6^ In particular, one-dimensional (1D) materials, including nanowires, nanorods, and nanotubes, have garnered considerable attention owing to their potential applications in nanoelectronics, nanodevices, nanocomposite materials, Li-ion batteries, and nanophotonics.^7−11^ Recent advances in the study of van der Waals (vdW) two-dimensional (2D) materials have led researchers to investigate the less-explored area of vdW 1D materials.^12−14^ vdW 1D materials are composed of molecular/atomic chains with strong intrachain covalent or ionic bonds and relatively weak interchain vdW interactions. This structural configuration, particularly the presence of vdW gaps in 1D materials, offers enhanced flexibility for structural manipulation and modification, and it is completely free from the limitations imposed by the strong covalent bonds in nominally three-dimensional (3D) materials.^12^

**Figure 1:**
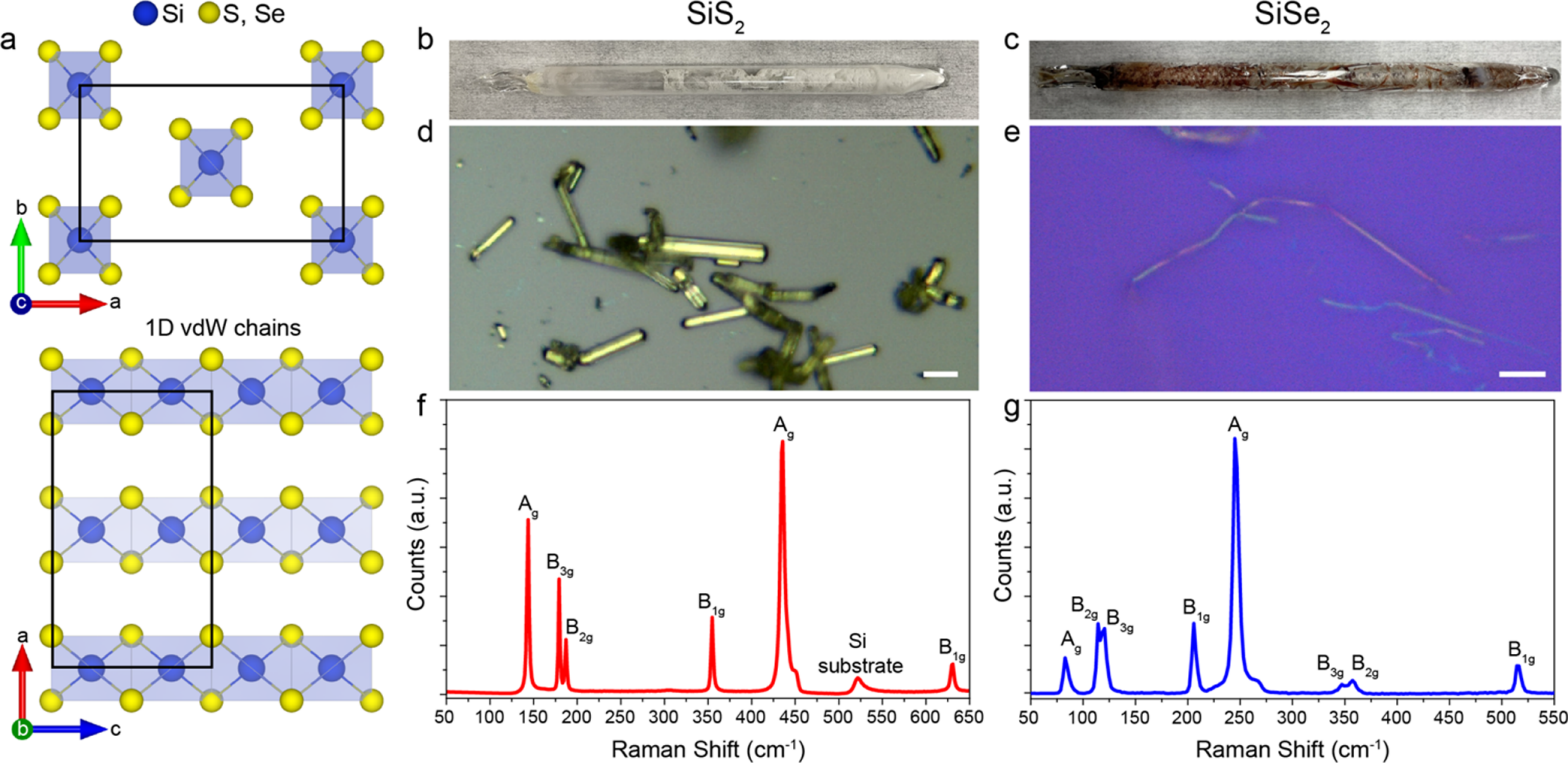
Bulk SiX_2_ synthesis and characterization. (a) Crystal structure of Bulk SiX_2_. The single chain consists of the edge-shared SiX_4_ tetrahedrons (silicon atoms blue, chalcogen atoms yellow). (b, c) Photos of the synthesized (b) SiS_2_ and (c) SiSe_2_ inside ampules. (d, e) Optical image of (d) SiS_2_ and (e) SiSe_2_ micro wires on SiO_2_/Si substrate. Scale bar: 20 and 5 μm, respectively. (f, g) Raman spectra of synthesized (f) SiS_2_ and (g) SiSe_2_.

Silicon or germanium dichalcogenides (AX_2_, A = Si or Ge and X = S or Se) are examples of vdW 1D chain structures with semiconducting properties. The bulk crystal structure of SiS_2_ and SiSe_2_ consists of edge-shared SiS_4_ and SiSe_4_ tetrahedral chains interconnected via vdW forces (Figure 1a).^15−17^ This atomic arrangement can lead to interesting physical phenomena as the thickness (number of chains) decreases. However, investigations of these materials at the single-chain level have been limited because of their poor environmental stability. Moreover, the formation of a ternary alloy structure (SiSSe) via the combination of SiS_2_ and SiSe_2_ illustrates the possibility of tailoring physical properties such as band gap by adjusting the alloy composition ratio.^18^ Although considerable research has focused on manipulating these properties via changes in the alloy composition, research on the single-chain limit is still lacking. Alloying in the single-chain limit may also be extended by incorporating analogous tetrahedral systems such as germanium chalcogenide tetrahedrons (GeS_4_ and GeSe_4_) to create quaternary alloy structures with diverse compositions. The realization and in-depth characterization of these alloy compositions can reveal their interesting material properties and application ranges by broadening the utility spectrum of vdW 1D materials.

The use of carbon nanotubes (CNTs) and boron nitride nanotubes (BNNTs) as reaction templates for encapsulation has proven highly effective for synthesizing various nanostructures.^19−23^ This innovative method enables the creation of a diverse array of vdW 1D materials by introducing various substances such as monoelements, perovskites, metal halides, carbides, and chalcogenides into the confined spaces of nanotubes.^24−36^ In particular, the small inner diameter of the nanotubes is advantageous for synthesizing 1D chain structures because it limits the radial growth of the encapsulated material. This geometric confinement effect of nanotubes has provided the opportunity for the emergence of crystal structures and properties not found in their bulk form.^37−40^ Furthermore, the protective sheaths formed by the nanotubes play a crucial role in shielding the encapsulated materials from degradation, thereby facilitating the investigation of various nanostructures. Consequently, the nanotube encapsulation method serves as a powerful and effective technique for the production of vdW 1D nanostructures and offers the potential for utilizing these materials in various technological areas.

Here, we demonstrate the stabilization of individual SiX_2_ chains in CNTs, including Ge-containing alloy compositions. Encapsulation in nanotubes significantly enhances the environmental stability of single chains, thereby enabling their comprehensive characterization. Atomic-resolution scanning transmission electron microscopy (STEM) reveals the detailed atomic structure, and we observe two distinct types of SiX_2_ chains. Moreover, we synthesize and characterize the Si_*x*_Ge_1–*x*_S_2(1–*y*)_Se_2*y*_ quaternary alloy chains, demonstrating their tunable semiconducting properties at the atomic-chain level. Density functional theory (DFT) calculations identify the electronic structures of these alloy chains and reveal their widely tunable electrical properties. This work provides possibilities for synthesizing and controlling the composition at the single-chain limit to tailor material properties.

## Results and Discussion

Bulk SiS_2_ and SiSe_2_ are directly synthesized using the melt-growth methods (see Methods section). Figure 1b,c shows the as-synthesized SiS_2_ and SiSe_2_ samples inside quartz ampules, respectively. The SiS_2_ samples exhibit small, thin, white powder-type crystals, whereas SiSe_2_ samples exhibit red-brown, needle-shaped crystals. The morphologies of the samples are characterized by optical microscopy (Figure 1d,e). SiS_2_ displays individual wires of a few micrometers, whereas SiSe_2_ shows a bundle of millimeter-sized crystals. Raman spectroscopy measurement is performed to identify the phase of SiS_2_ and SiSe_2_. Figure 1f,g shows the Raman spectra of the crystalline SiS_2_ and SiSe_2_, respectively. The Raman peaks (144, 180, 187, 355, 436, and 630 cm^–1^) from SiS_2_ and (83, 114, 120, 206, 245, 346, 357, and 515 cm^–1^) from SiSe_2_ are obtained. The observed highly intensive and narrow peak at 437.4 cm^–1^ for SiS_2_ and 244 cm^–1^ for SiSe_2_ corresponds to the *A*_g_ symmetric stretching mode of SiX_4_, which suggests that the synthesized product is highly crystalline.^16,17^ All of the observed Raman peaks of SiS_2_ and SiSe_2_ are assigned and matched well with previously reported Raman results.^18^

The SiS_2_ and SiSe_2_ samples exhibit high reactivity to ambient exposure, particularly to air and moisture, which triggers the formation of toxic gases such as H_2_S and H_2_Se.^18^ The surface of these materials degrades upon exposure, resulting in the formation of bubbles, as shown in Figure S1. Additionally, the Raman spectrum of bulk SiX_2_ distinctly shows a decrease in intensity following exposure to air. This high reactivity poses significant challenges in extending the production of SiS_2_ and SiSe_2_ to the single-chain limit and in further characterization.

**Figure 2:**
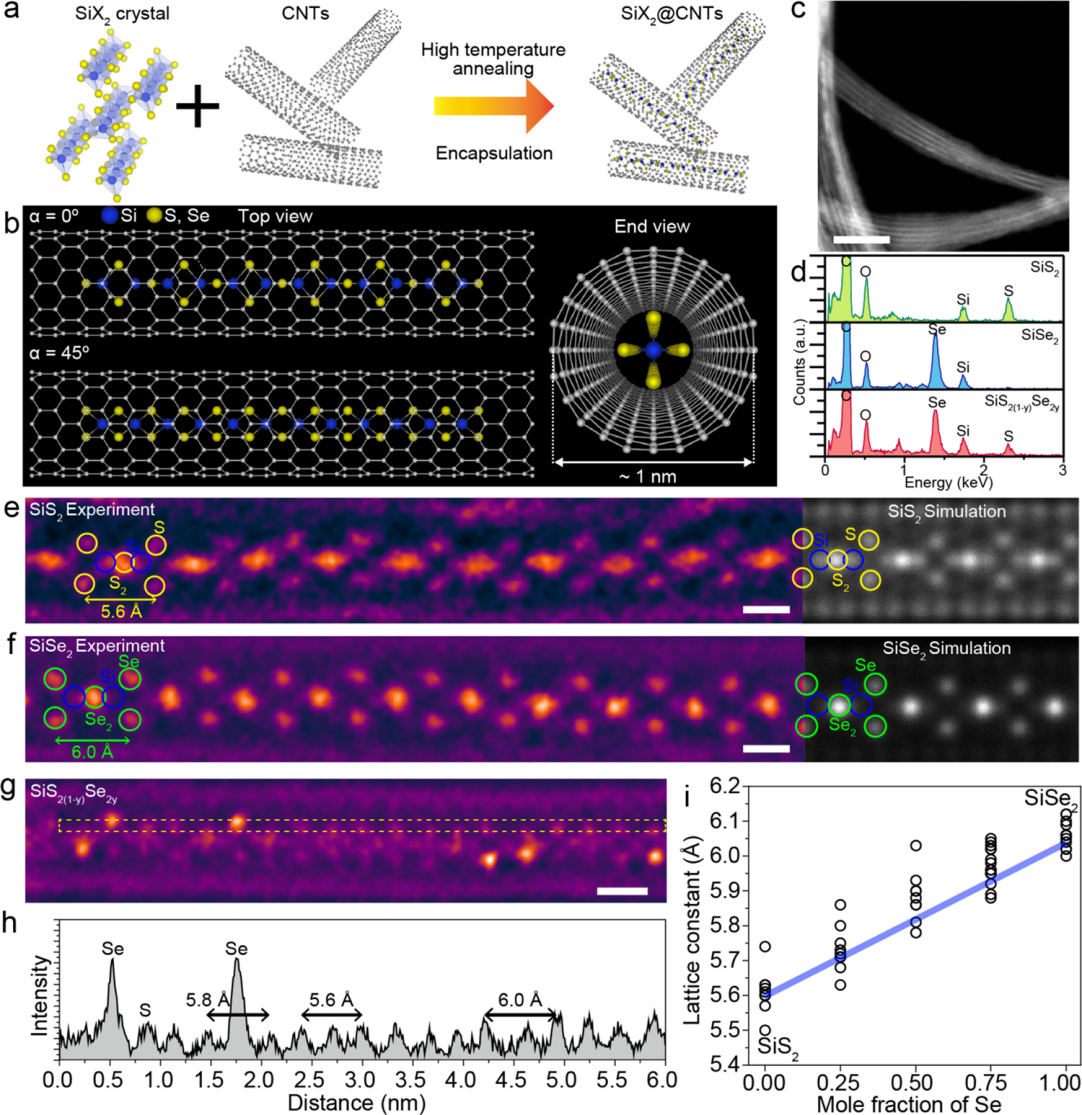
Isolation type-1 1D SiX_2_ single chain inside nanotube. (a) Schematic of isolation of the 1D SiX_2_ vdW chains via nanotube encapsulation. (b) Atomic model of type-1 SiX_2_ single chain inside nanotube. (c) Low-magnification image of SiSe_2_ encapsulated nanotube. Scale bar: 20 nm. (d) EDS spectra of SiS_2_, SiSe_2_, and SiS_2(1–*y*)_Se_2*y*_ alloy chains inside nanotubes. (e–g) Atomic-resolution STEM images of type-1 (e) SiS_2_, (f) SiSe_2_, and (g) SiS_2(1–*y*)_Se_2*y*_ alloy chain. Simulated STEM images of SiS_2_ and SiSe_2_ are shown on the right. Scale bar: 0.5 nm. (h) ADF intensity line profile of SiS_2(1–*y*)_Se_2*y*_ alloy chain inside the yellow square in panel (f). (i) Experimentally measured lattice constant versus Se fraction in the SiS_2(1–*y*)_Se_2*y*_ alloy sample.

We utilized a nanotube reaction vessel to isolate SiX_2_ single chains and protect them from degradation (Figure 2a). SiX_2_ chains in nanotube samples are synthesized by directly sublimating the SiX_2_ precursors with open-ended nanotubes in sealed ampules, as described in the Methods section. Encapsulating SiX_2_ in the nanotubes enables isolation of the material at the single-chain level and provides a high degree of environmental stability, thereby facilitating detailed characterization. Figure 2b illustrates the overall structure of the 1D SiX_2_ single chain within the nanotube. The synthesized SiX_2_ encapsulated nanotubes exhibit high filling fractions (approximately 90%) with chain lengths extending to hundreds of nanometers, as shown in Figures 2c and S2. The atomic composition ratio of SiS_2_ and SiSe_2_ is confirmed by energy-dispersive spectroscopy (EDS), which reveals a 1:2 atomic percentage ratio of Si to S or Se (Figure 2d). We also synthesize a SiS_2(1–*y*)_Se_2*y*_ alloy chain inside the nanotubes, and EDS characterization confirms the coexistence of the S and Se from the sample. The average composition ratio of the alloy sample is SiS_0.8_Se_1.2_, as determined by EDS quantification.

The detailed atomic structure of the synthesized SiX_2_ encapsulated nanotube samples is investigated using annular dark-field (ADF) aberration-corrected STEM. Figure 2e,f shows the atomic-resolution ADF-STEM image of SiS_2_ and SiSe_2_ single chain encapsulated in a nanotube with an inner diameter of 1 nm. The image contrast of ADF-STEM strongly depends on the atomic number (*Z*); thus, S (*Z*_s_ = 16) and Se (*Z*_se_ = 34) exhibit a higher intensity than Si (*Z*_si_ = 14). The simulated STEM images (right side in Figure 2e,f) are generated using the identified atomic structure, which matches well with the experimentally observed result. The observed 1D single-chain structure of SiS_2_ and SiSe_2_ in the nanotubes exhibits 1D chain structures with edge-shared tetrahedrons, which are identical to the single chains present in the bulk material, which we refer to as type-1 structure.^40^ The atomic-resolution images of the 45° rotated SiS_2_ and SiSe_2_ chain structures within nanotubes also well match with our expected structure (Figure S3). The measured lattice constants of SiX_2_ chains along the chain directions are 5.6 Å for SiS_2_ and 6.0 Å for SiSe_2_, which are in good agreement with the previously reported chain distance from the bulk SiX_2_ crystal structure.^15^

The SiS_2(1–*y*)_Se_2*y*_ ternary alloy sample maintains an identical chain structure within the nanotubes. Figure 2g shows an atomic-resolution ADF-STEM image of the type-1 SiS_2(1–*y*)_Se_2*y*_ alloy chain in a nanotube. Because of the higher atomic number of Se (*Z*_se_ = 34), the Se atomic positions are brighter than the S or Si atomic positions. Figure 2h shows the intensity line profile from the regions marked in Figure 2g, which clearly shows the intensity variation between Se and S. The larger atomic radius of Se compared to that of S leads to a modification in the lattice constant; for instance, the original lattice constant of SiS_2_, 5.6 Å, increases to 5.8 Å with the substitution of one S atom for Se. As the mole fraction of Se increased, the lattice constant increased linearly and reached 6.0 Å, which is the lattice constant of SiSe_2_. Figure 2i shows the experimentally measured lattice constant as a function of the Se fraction in SiSSe, showing linear behavior following Vegard’s law. The substitution of S and Se in the SiSSe lattice may induce distortion and residual strain owing to the size mismatch between S and Se atoms. Despite the random distribution of chalcogenide atoms (S or Se) along the chains, they continue to preserve the edge-shared tetrahedral chain structure within the 1 nm diameter of the nanotubes. All of the experimentally observed STEM images are in good agreement with the simulated STEM images, as shown in Figures S4–S6.

The internal diameter of the nanotube plays a crucial role in determining the confined structure. In a prior investigation, we discovered an alternative chain structure of GeX_2_ in nanotubes with diameters ranging from 1.0 to 1.2 nm, called type-2.^40^ This type-2 chain structure, featuring tetrahedral sharing edges and corners, deviates from the bulk GeX_2_ structure. Similarly, we observed an identical type-2 SiX_2_ chain structure in nanotubes with diameters ranging from 1.0 to 1.2 nm. Figure S7 shows the experimentally observed type-2 SiX_2_ single chains inside the nanotubes, with atomic structures identical to those previously observed in the GeX_2_ system.^40^ Both the experimental and simulated STEM images of type-2 SiX_2_ along various projection directions exhibit excellent agreement (Figures S8 and S9).

Other structures are also observed in the nanotubes with different diameters, as shown in Figure S10. Nanotubes with diameters less than 0.9 nm exhibit S or Se (even mixed case) single atomic-chain structures inside, whereas those with inner diameters larger than 1.2 nm result in the formation of multiple chains of SiX_2_. These findings align with the previously studied diverse structures inside nanotubes of various diameters.^21,22,24,37,41−43^ Our observations lead to the conclusion that the type-1 chain structure is stabilized in nanotubes with diameters ranging from 0.9 to 1.0 nm, whereas the type-2 chain structure is stabilized in nanotubes with diameters ranging from 1.0 to 1.2 nm (Figure S11).

**Figure 3:**
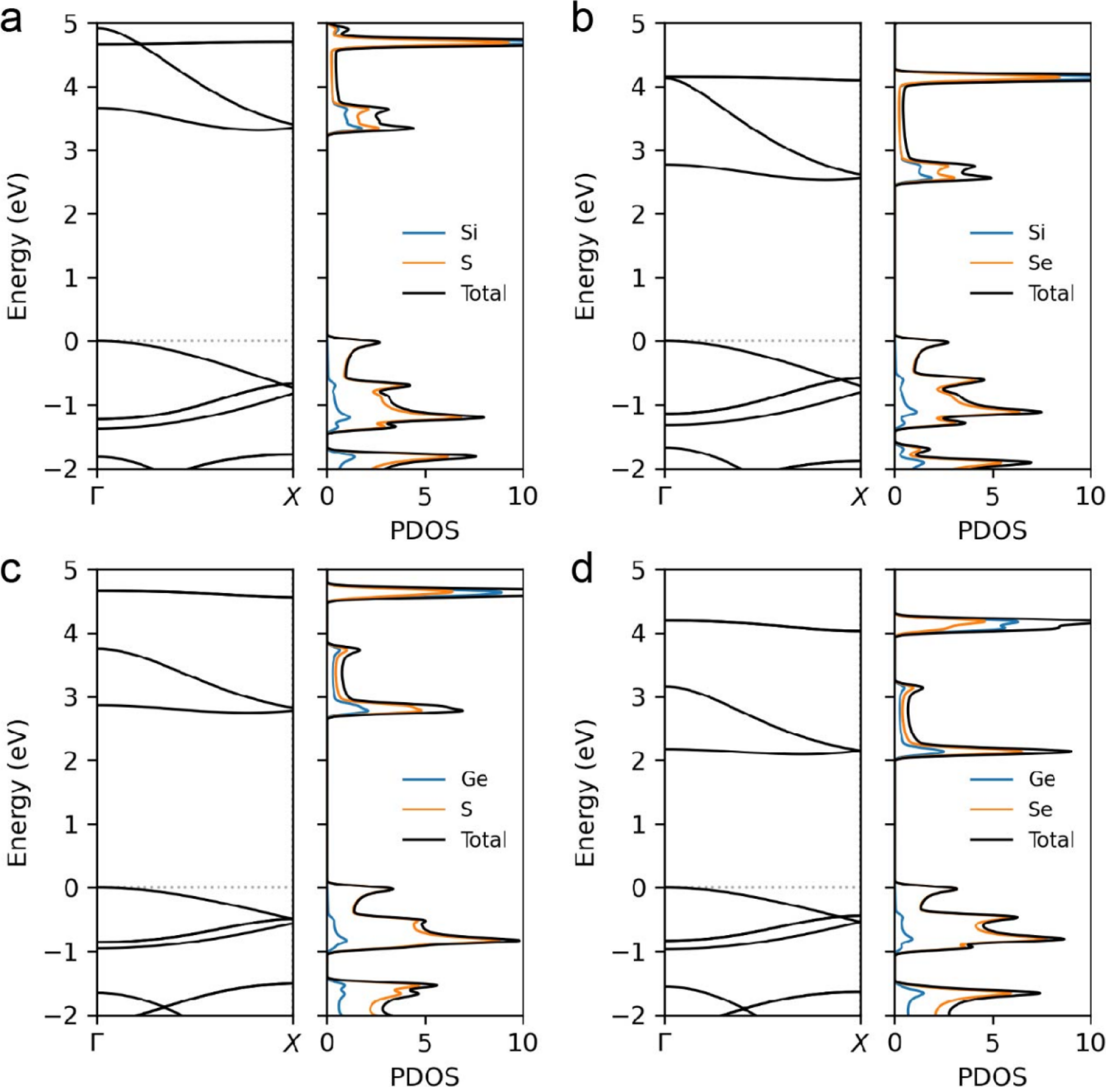
Calculated band structures and projected density of states (PDOS) of type-1 single chain. (a, b) SiX_2_ (X = S and Se) and (c, d) GeX_2_ for comparison. The valence bands mostly consist of chalcogen atomic orbitals, whereas the conduction bands states are distributed over all atoms.

We theoretically investigate the atomic structural and electronic properties of SiX_2_ single chains using first-principles DFT calculations. The calculated lattice parameter along the axial direction is 5.68 and 6.00 Å for type-1 SiS_2_ and SiSe_2_, respectively, which are in good agreement with the experimental measurements. Figures 3 and S12 show the calculated electronic structures of SiX_2_ chains as well as GeX_2_ chains for comparison. All of the type-1 chains are indirect-gap semiconductors with the band gap of 3.31 and 2.53 eV for SiS_2_ and SiSe_2_, respectively. Conversely, the type-2 chains are identified as direct-gap semiconductors, with band gaps of 1.85 eV for SiS_2_ and 1.22 eV for SiSe_2_. In a single chain of SiX_2_, the elimination of interactions between different chains leads to the absence of band dispersion in the plane perpendicular to the chains, resulting in a larger band gap than that of bulk SiX_2_.^44^ The valence bands predominantly consist of chalcogen atomic orbitals, while the conduction bands are spread across all atomic types. In our previous study, we found that CNT encapsulation did not markedly modify the atomic and electronic structures of GeX_2_ chains, provided that there was no significant charge transfer between the GeX_2_ chains and the CNTs.^40^ Based on these observations, we assumed that the SiX_2_ chains would exhibit similar behavior when encapsulated in CNTs. Therefore, all encapsulated chains, both SiX_2_ and GeX_2_, retain their semiconducting states.

**Figure 4:**
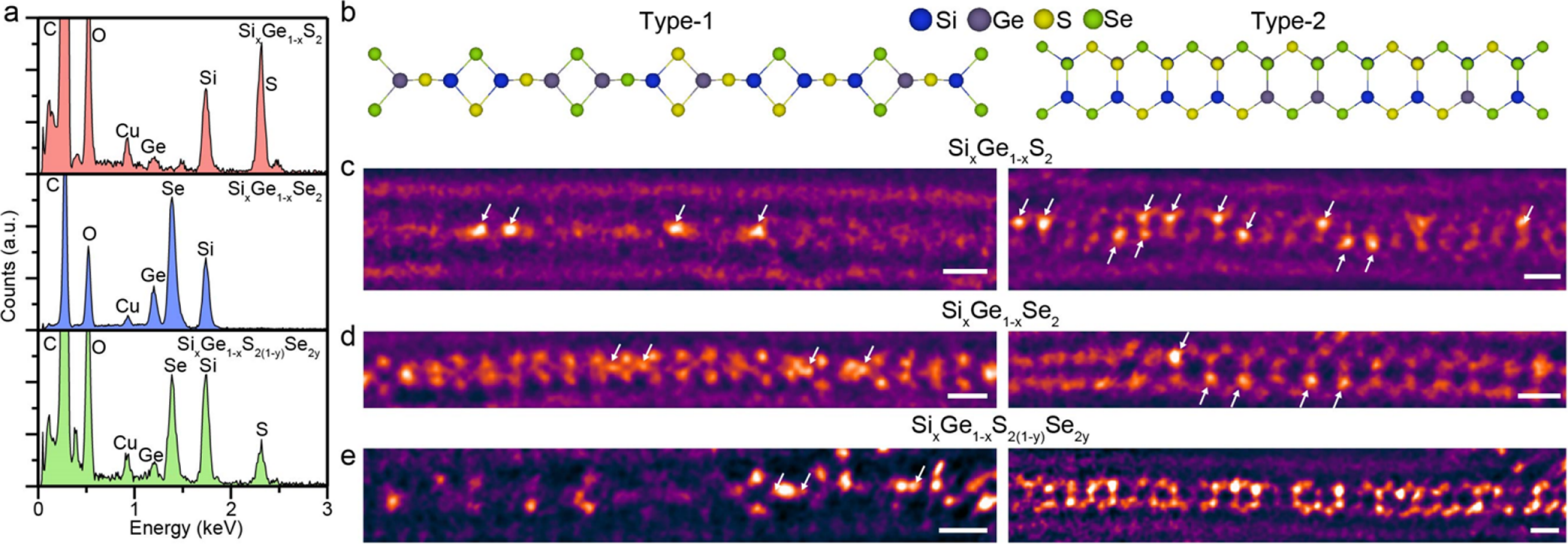
1D Si*_x_*Ge_1–_*_x_*S_2(1–*y*)_Se_2*y*_ alloy chain inside nanotube. (a) EDS spectra of Si_*x*_Ge_1–*x*_S_2_, Si_*x*_Ge_1–*x*_Se_2_, and Si_*x*_Ge_1–*x*_S_2(1–*y*)_Se_2*y*_ alloy chains inside nanotubes. (b) Atomic model of (left) type-1 and (right) type-2 Si_*x*_Ge_1–*x*_S_2(1–*y*)_Se_2*y*_ alloy chain structure. (c–e) Atomic-resolution STEM images of the (c) Si_*x*_Ge_1–*x*_S_2_, (d) Si_*x*_Ge_1–*x*_Se_2_, and (e) Si_*x*_Ge_1–*x*_S_2(1–*y*)_Se_2*y*_ alloy single chain inside nanotube. Scale bar: 0.5 nm. White arrows indicate the atomic position of Ge.

Extending the aforementioned findings for SiX_2_ and the previously studied GeX_2_, we study the 1D Si_*x*_Ge_1–*x*_S_2(1–*y*)_Se_2*y*_ quaternary single chains inside nanotubes with a different alloy composition, as shown in Figure 4. We synthesized various combinations of Si_*x*_Ge_1–*x*_S_2(1–*y*)_Se_2*y*_ alloy chains inside nanotubes using mixed SiX_2_ and GeX_2_ precursors. The synthesized alloy samples are confirmed by EDS in STEM from a bundle of nanotubes. The EDS spectra exhibit well-defined peaks for each element (Si, Ge, S, and Se) in the Si_*x*_Ge_1–*x*_S_2_, Si_*x*_Ge_1–*x*_Se_2_, and Si_*x*_Ge_1–*x*_S_2(1–*y*)_Se_2*y*_ alloy samples (Figure 4a). From the EDS quantitative analysis, the exemplary synthesized alloy samples are confirmed to be Si_0.7_Ge_0.3_S_2_, Si_0.8_Ge_0.2_Se_2_, and Si_0.9_Ge_0.1_S_0.4_Se_1.6_. Additionally, electron energy loss spectroscopy (EELS) is utilized to reveal the presence of Si, S, and Se in the Si_*x*_Ge_1–*x*_S_2(1–*y*)_Se_2*y*_ alloy within a single isolated nanotube (Figure S13). The Ge core-loss peak was not detected in the isolated nanotube sample because of the low Ge concentration in the quaternary alloy sample.

Given that both SiX_2_ and GeX_2_ form the same type of chain structure inside the nanotube, we expect the formation of type-1 (left side) and type-2 (right side) Si_*x*_Ge_1–*x*_S_2(1–*y*)_Se_2*y*_ alloy chains, as shown in Figure 4b. Via successful synthesis and atomic-resolution STEM imaging, we observe both type-1 and type-2 chain structures in the Si_*x*_Ge_1–*x*_S_2_, Si_*x*_Ge_1–*x*_Se_2_, and Si_*x*_Ge_1–*x*_S_2(1–*y*)_Se_2*y*_ samples (Figure 4c–e). In the ADF-STEM imaging, the Ge atomic positions exhibit a brighter contrast compared to those of Si and S, enabling the clear identification of Ge atoms in the Si_*x*_Ge_1–*x*_S_2_ alloy sample. In Si_*x*_Ge_1–*x*_Se_2_ and Si_*x*_Ge_1–*x*_S_2(1–*y*)_Se_2*y*_, Se appears to be the brightest element. Contrast variations may occur based on the rotation of the chain structure and overlapping elements. For example, in 30° rotated type-2 Si_*x*_Ge_1–*x*_S_2(1–*y*)_Se_2*y*_ (Figure 4e), the brightest atomic position corresponds to Ge + Se, and the darkest atomic position corresponds to Si + S. The STEM image simulations of both type-1 and type-2 are in good agreement with the experimentally observed image contrast variations (Figures S14–S17). Our study successfully demonstrates the synthesis of quaternary alloy chains at the single-chain limit via nanotube encapsulation.

**Figure 5:**
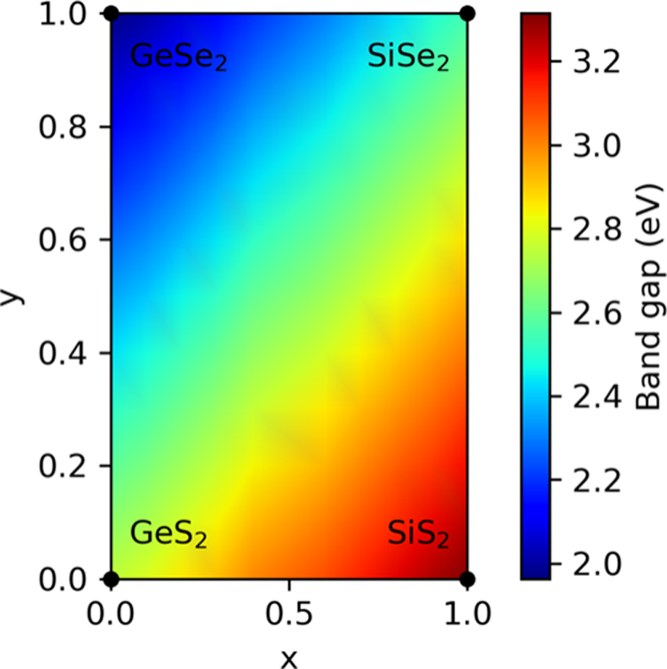
Band gap variation of the type-1 Si*_x_*Ge_1–*x*_S_2(1–*y*)_Se_2*y*_ alloy chain as a function of composition. For a given composition, atomic structures and lattice parameters are linearly interpolated, and electronic structures are calculated by using the virtual crystal approximation.

Finally, we discuss the composition-dependent electronic structures of quaternary alloy chains. Figure 5 shows the calculated band gaps of type-1 Si_*x*_Ge_1–*x*_S_2(1–*y*)_Se_2*y*_ alloy chains with different compositions. For a given composition, the atomic structures are linearly interpolated from the pure compositions of the chains: GeS_2_ at (*x* = 0, *y* = 0), GeSe_2_ at (*x* = 0, *y* = 1), SiS_2_ at (*x* = 1, *y* = 0), and SiSe_2_ at (*x* = 1, *y* = 1). We then calculate the electronic structure of the alloy chains using the virtual crystal approximation (VCA). The lower band gap regions, which are the blue regions in the figure, correspond to Ge-rich and Se-rich alloys, whereas the higher band gap regions in red correspond to Si-rich and S-rich alloys. This suggests that widely tunable band gaps, which are critical for atomic-scale optoelectronic applications, can be achieved by altering the ratios of Si, Ge, S, and Se in the single-chain limit.

## Conclusions

In conclusion, we report the stabilization of 1D SiX_2_ single chains via nanotube encapsulation. Our findings reveal that nanotube encapsulation stabilizes the materials and induces modifications in the chain structure of the confined material. Furthermore, we successfully demonstrate the synthesis and control of the composition of Si_*x*_Ge_1–*x*_S_2(1–*y*)_Se_2*y*_ quaternary alloys at the single-chain level. Electronic structure calculations reveal that the band gap of these semiconducting alloy chains is highly adjustable and dependent on both the structural and the compositional ratio. These findings provide further groundwork for the study of low-dimensional and confinement-stabilized materials in nanotubes and offer opportunities for future research and applications in various fields.

## Methods

### Materials

To synthesize SiS_2_ and SiSe_2_ precursors, we mixed silicon (99.999% Alfa Aesar) with sulfur (99.999% Alfa Aesar) or selenium (99.999% Alfa Aesar) in an atomic ratio of 1:2. The mixture (1g) was then sealed in a 10 mm diameter and 15 cm long ampule under high vacuum (∼10^–6^ Torr) and placed in a horizontal one-zone furnace with the hot end at 850 °C for 5 days. After synthesis, the materials were extracted inside an Ar-filled glovebox to minimize oxidation. The GeS_2_ and GeSe_2_ powders were purchased from Ossila.

### Growing SiX_2_ and Alloy Chains @ Nanotube

CNTs were purchased from Sigma-Aldrich (single-walled: 704113) and Cheap Tubes (90% Single Walled-Double Walled (SW-DW) CNTs) and were annealed in air at 510 °C for 15 min to open the end caps. The open-ended CNTs (approximately 3 mg) were mixed with 20 mg of the precursor material and sealed in a 6 mm diameter and 15 cm long quartz ampule under a high vacuum (∼10^–6^ Torr). The sealed ampule was then heated to 1000 °C in a single zone box furnace and kept there for 5 days before being cooled to room temperature for over 1 day. The synthesized materials (SiX_2_@nanotube) were dispersed in isopropanol using a bath sonicator for 15 min and drop-cast onto lacey carbon Transmission Electron Microscopy (TEM) grids for STEM characterization. The alloy samples were also synthesized according to the procedure described above.

### Optical Characterizations

Optical microscopy images were obtained using a Leica-DM750M inside an N_2_-filled glovebox. Raman measurements were performed by using a Renishaw inVia confocal Raman microscope with a 514 nm laser under ambient conditions.

### TEM/STEM Imaging and Simulations

TEM and STEM images were acquired using a double spherical (Cs) aberration-corrected JEOL ARM-200F operated at 80 kV. For atomic-resolution STEM imaging, a microscope was used with a 23 or 30 mrad convergence angle and collection semiangles ranging from 40–160 mrad.

Atomic-resolution STEM image simulations were performed by using MacTempas software based on multislice calculations. The simulation parameters were similar to the experimental parameters (e.g., a probe semiangle of 23 or 30 mrad, 0.05 Å/pixel sampling, and 20 frozen phonon calculations) for each simulation. Image analysis and processing were performed by using ImageJ software. Poisson noise was added to the simulated STEM images to match the experimental results.

### EELS Characterizations

The STEM-EELS experiments were performed in a Nion HERMES microscope equipped with a C3/C5 corrector at an accelerating voltage of 60 kV. The beam convergence semiangle was 32 mrad, and the collection semiangle for EELS was 75 mrad, with the EELS aperture out.

### Calculations

We performed first-principles DFT calculations as implemented in SIESTA.^45^ We used the Perdew–Burke–Ernzerhof (PBE) functional,^46^ norm-conserving pseudopotentials,^47^ and a localized pseudoatomic orbital basis. van der Waals interactions were included within the Grimme-D2 scheme.^48^ A real-space mesh cutoff of 500 Ry was used. We used a 25 Å thick cell along the transverse vacuum direction. The primitive Brillouin zone of the isolated chains was sampled by 12 *k* points. The atomic positions were optimized with a force threshold of 0.01 eV/Å. In the alloy calculations, we used the virtual crystal approximation by mixing the pseudopotentials of Si with Ge and S with Se, respectively.

## Supporting Information Available

Optical images of the SiS_2_ under ambient exposure; extra STEM characterizations of samples; including experimental atomic-resolution images and EELS characterizations; simulated STEM images; and DFT calculations for the electronic structure of type-2 SiX_2_ and GeX_2_ (PDF)
