# Supplementary material for: SiX2 (X = S, Se) Single Chains and (Si–Ge)X2 Quaternary Alloys
Source: ACS Nano. 2024 Jun 26;18(27):17882–9. doi: 10.1021/acsnano.4c04184 (PMC11238589; doi:10.1021/acsnano.4c04184)
Supplement: Supplementary file 1 — nn4c04184_si_001.pdf [file nn4c04184_si_001.pdf]

Supporting Information for

SiX_2 (X=S, Se) Single Chains and (Si-Ge)X_2 Quaternary Alloys

Yangjin Lee[1,2,3,4,†,], Young Woo Choi[1,2,†], Linxuan Li[5], Wu Zhou[5], Marvin L. Cohen[1,2], Kwanpyo Kim[3,4,*], and Alex Zettl[1,2,6,*]*

[1]Department of Physics, University of California at Berkeley, Berkeley, California 94720, United States

[2]Materials Sciences Division, Lawrence Berkeley National Laboratory, Berkeley, California 94720, United States

[3]Department of Physics, Yonsei University, Seoul 03722, Korea

[4]Center for Nanomedicine, Institute for Basic Science, Seoul 03722, Korea

[5]School of Physical Sciences and CAS Key Laboratory of Vacuum Physics, University of Chinese Academy of Sciences, Beijing 100049, People's Republic of China

[6]Kavli Energy NanoSciences Institute at the University of California at Berkeley, Berkeley, California 94720, United States

† These authors contributed equally to this work.

*Address correspondence to Y.L (yangjinlee@yonsei.ac.kr), K.K. (kpkim@yonsei.ac.kr) and A.Z. (azettl@berkeley.edu)

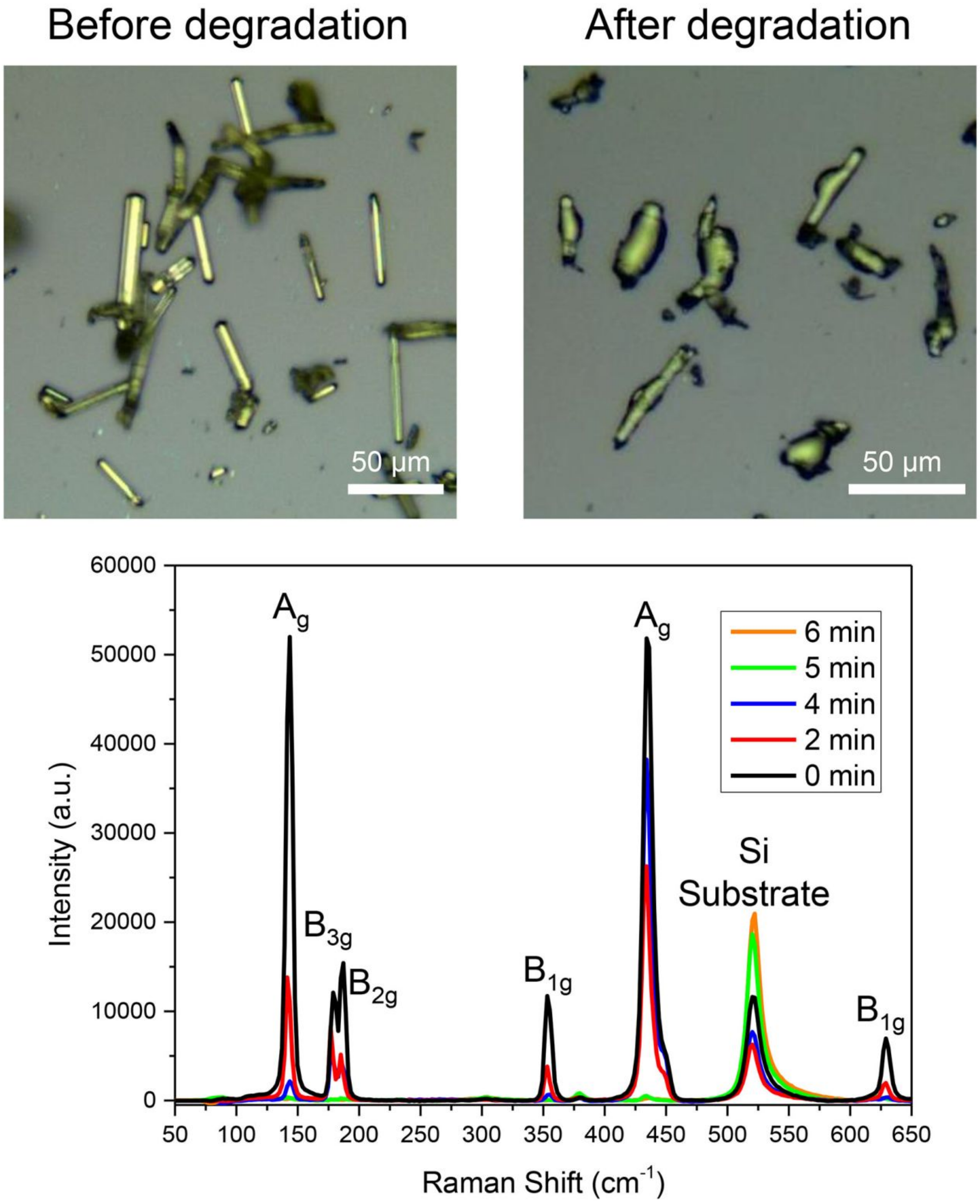

Supporting Figure S1. Optical images and Raman spectra showing the degradation of SiS_2 under ambient exposure.

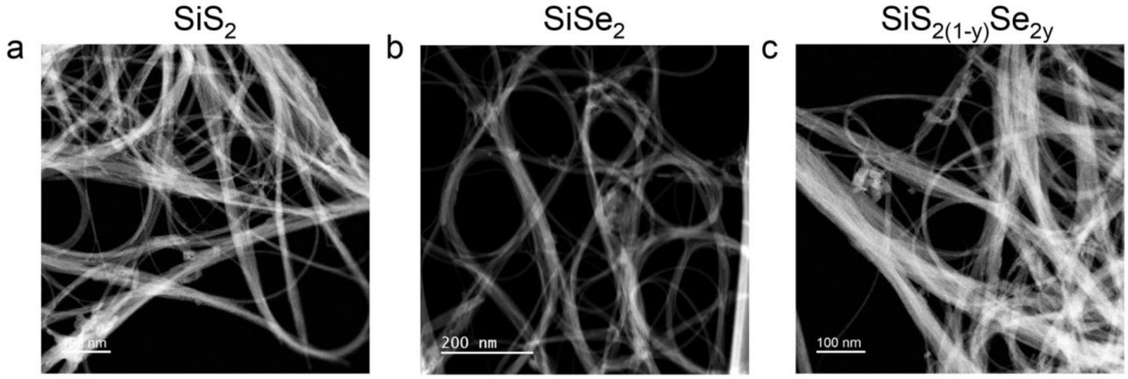

Supporting Figure S2. Low-magnification STEM images of SiS_2, $SiSe_2$, and $SiS_{2(1-y)}Se_{2y}$ alloy chains inside nanotubes.

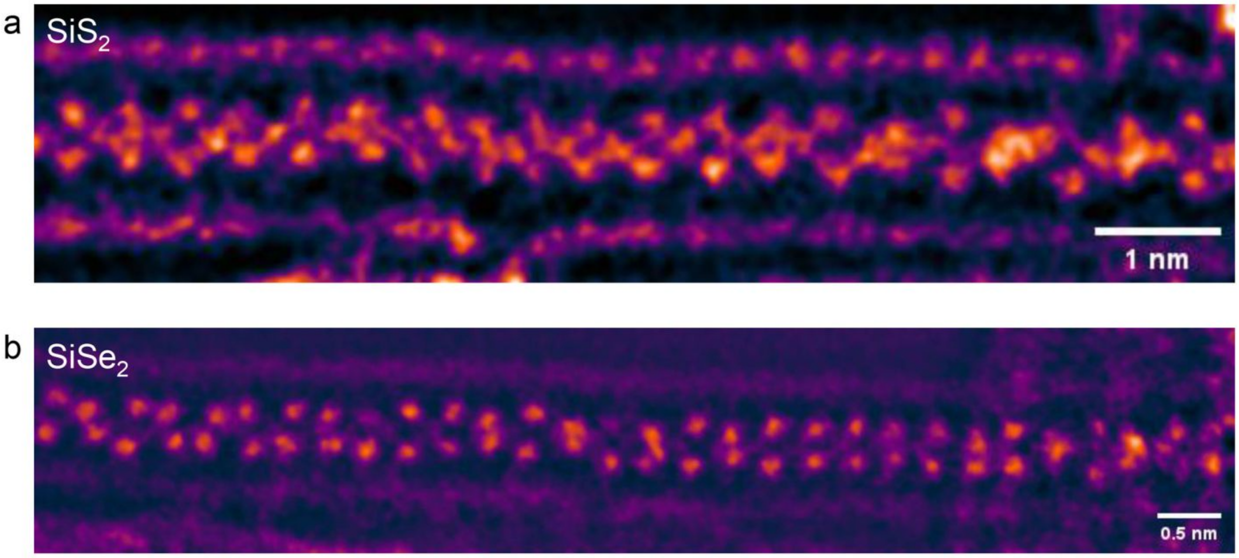

Supporting Figure S3. Experimental STEM images of type-1 SiS_2, and $SiSe_2$ at the different viewing direction.

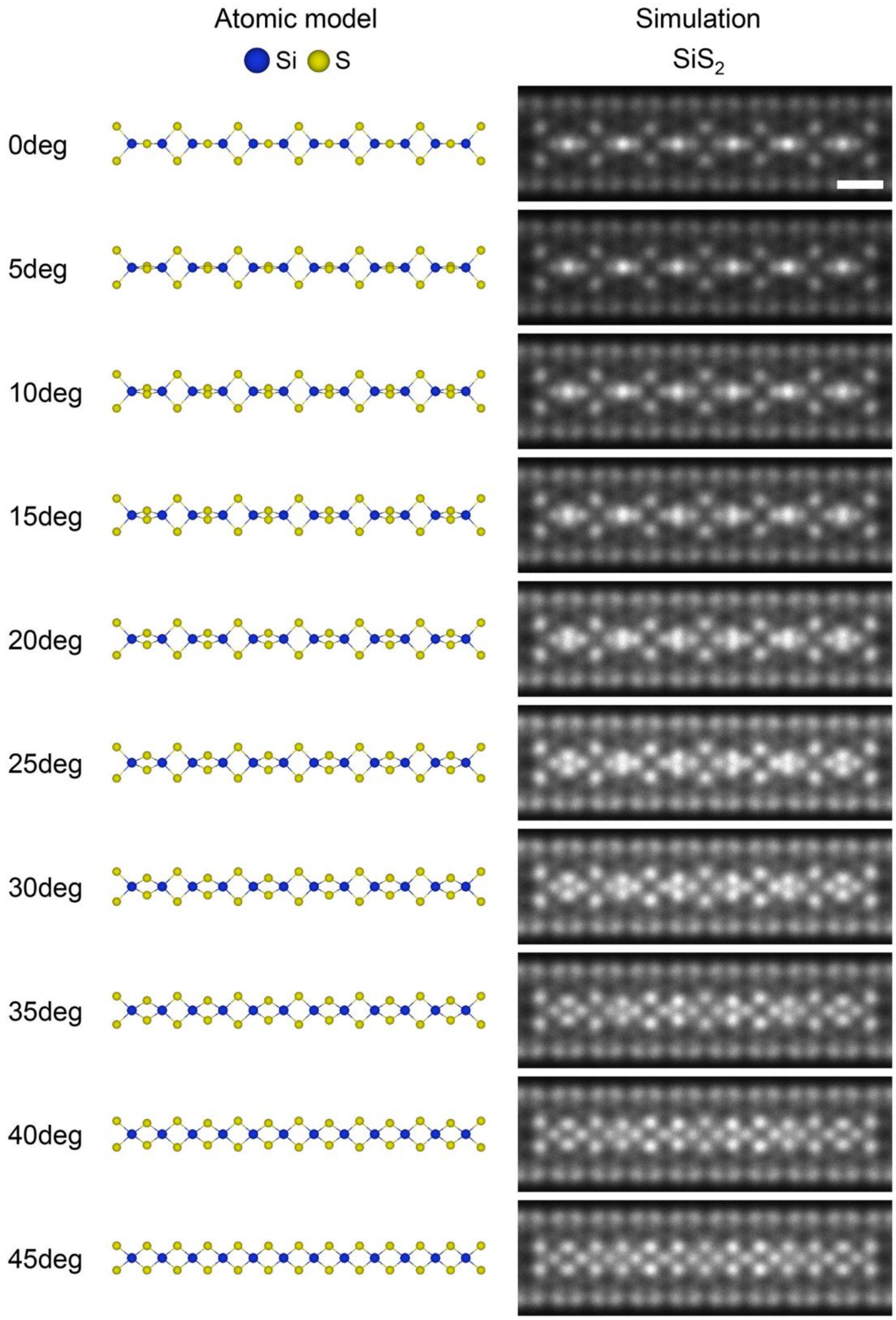

Supporting Figure S4. Simulated STEM images of type-1 SiS_2 with various rotation angles. Scale bar: 0.5 nm.

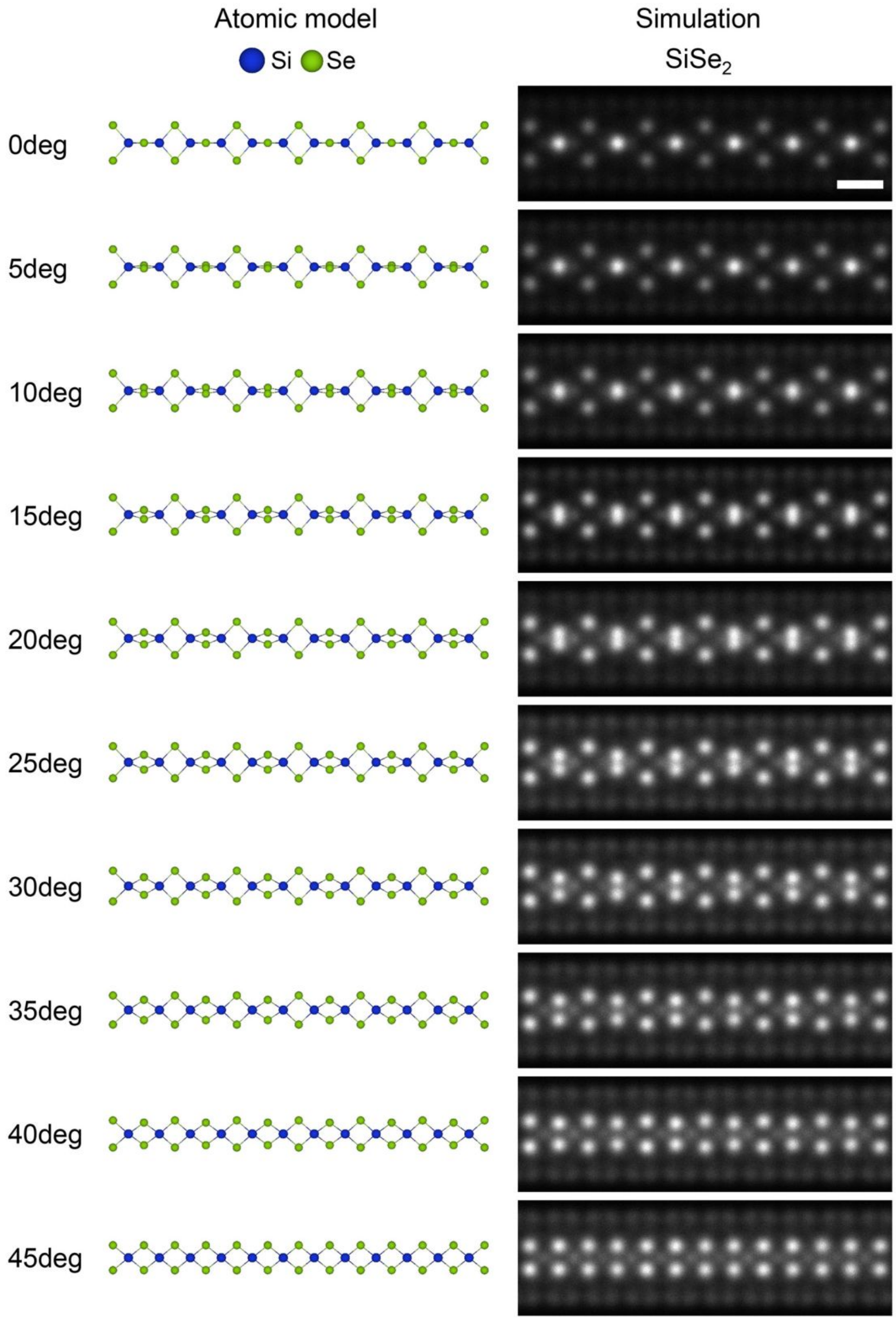

Supporting Figure S5. Simulated STEM images of type-1 $SiSe_2$ with various rotation angles. Scale bar: 0.5 nm.

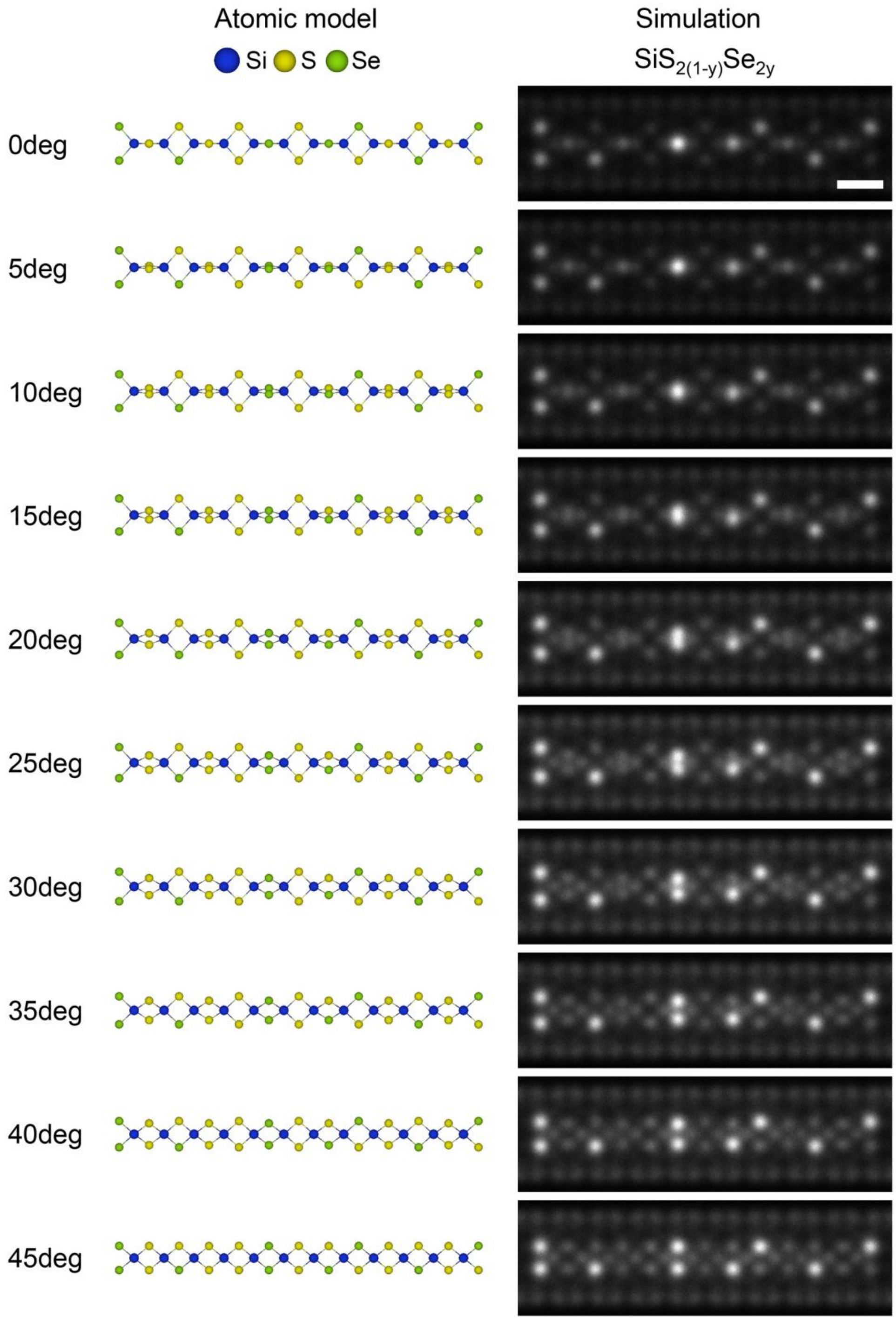

Supporting Figure S6. Simulated STEM images of type-1 $SiSSe_2$ with various rotation angles. Scale bar: 0.5 nm.

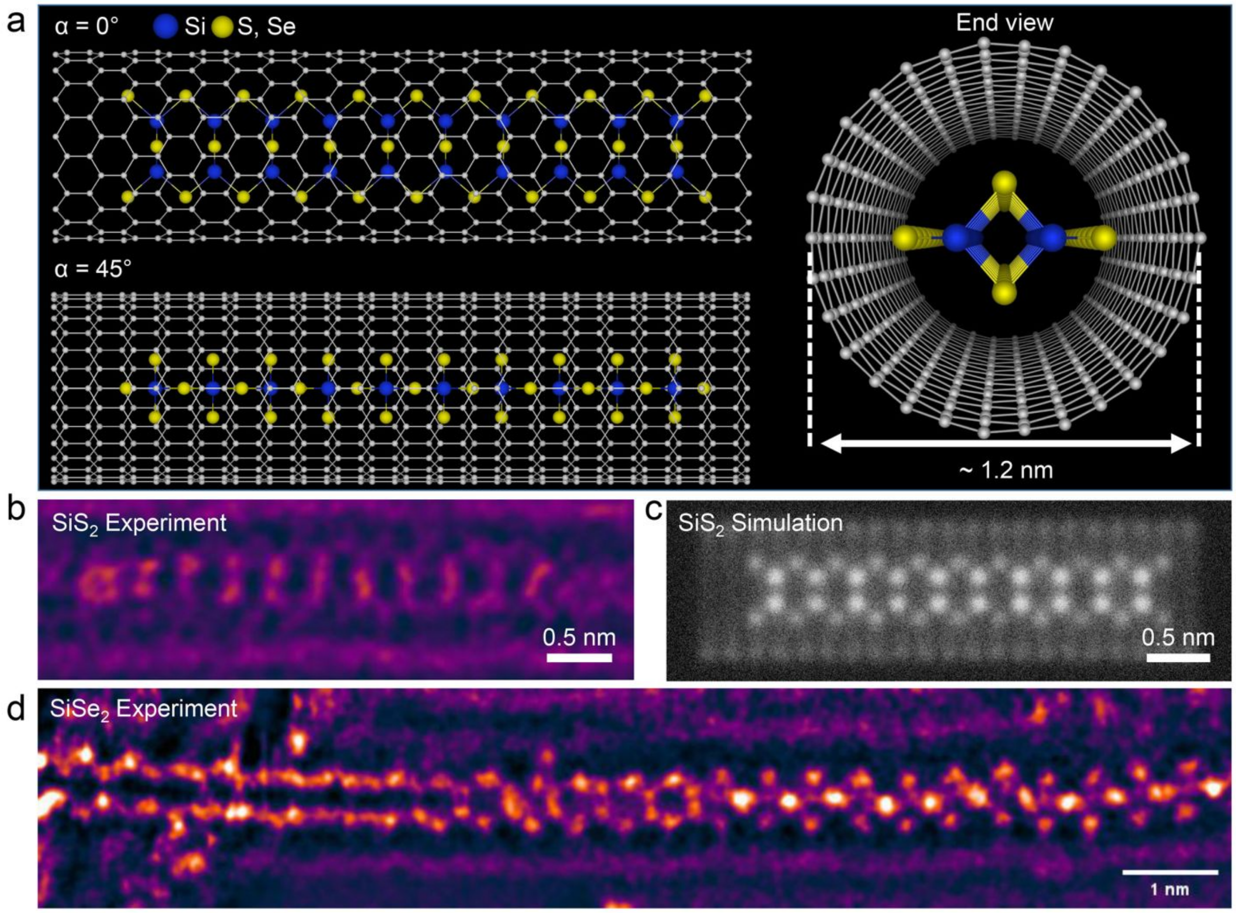

Supporting Figure S7. Type-2 SiS_2 and $SiSe_2$ inside nanotube. (a) atomic model of type-2 SiX_2 structure. (b) Experimental and (c) simulated STEM image of SiS_2 inside nanotube. (d) Experimental STEM image of $SiSe_2$ inside nanotube. Scale bar: 1nm.

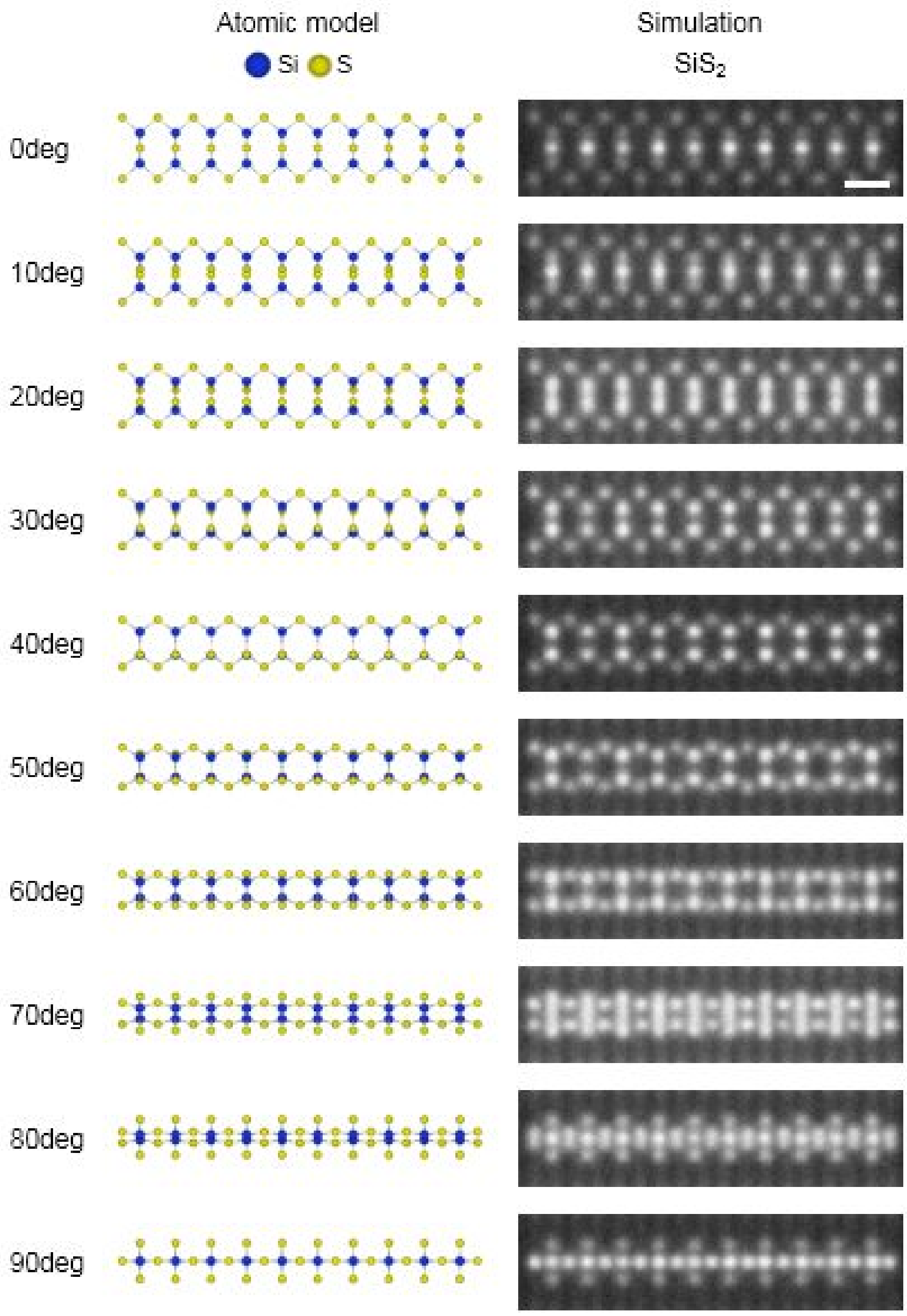

Supporting Figure S8. Simulated STEM images of type-2 SiS_2 with various rotation angles. Scale bar: 0.5 nm.

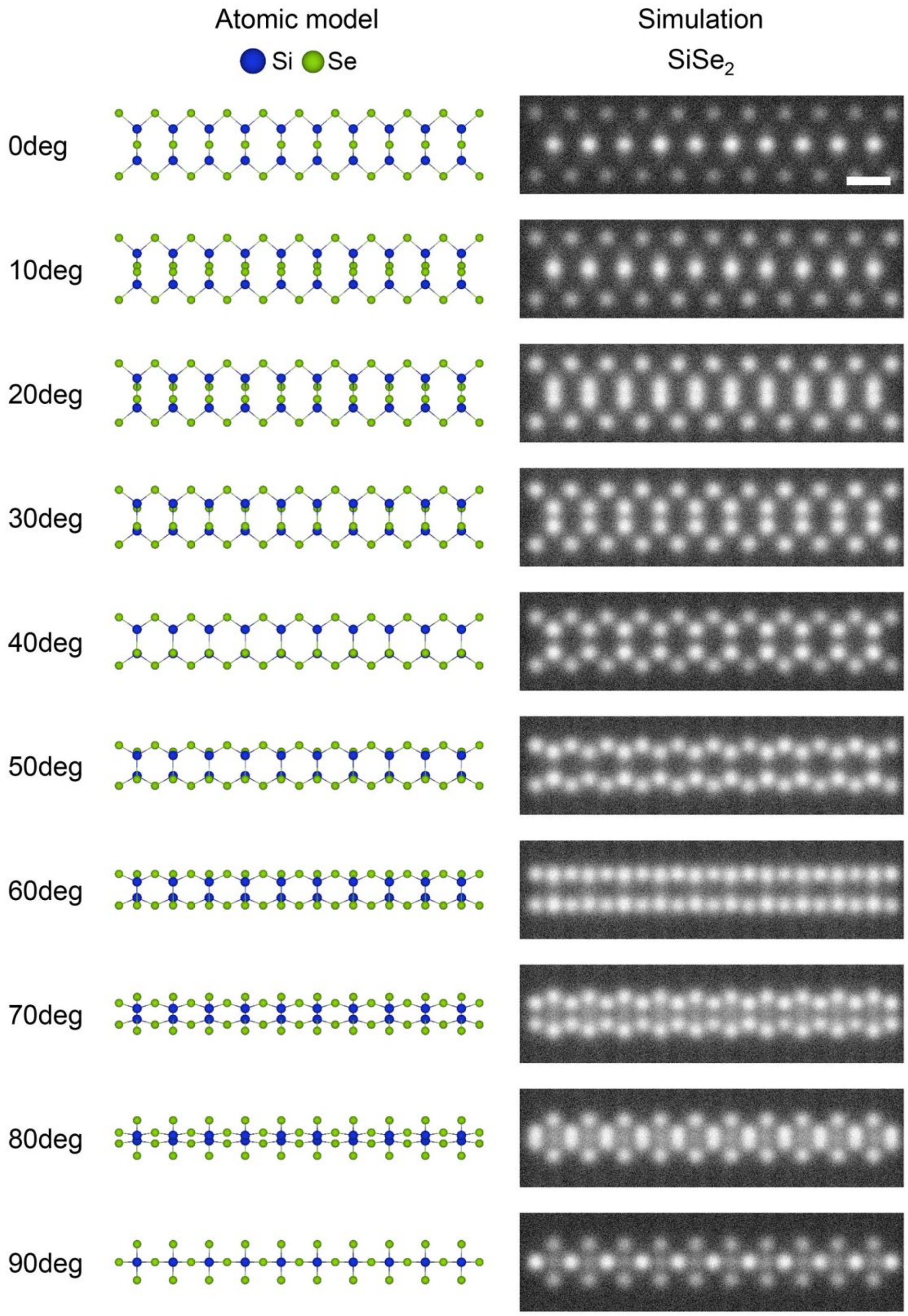

Supporting Figure S9. Simulated STEM images of type-2 $SiSe_2$ with various rotation angles. Scale bar: 0.5 nm.

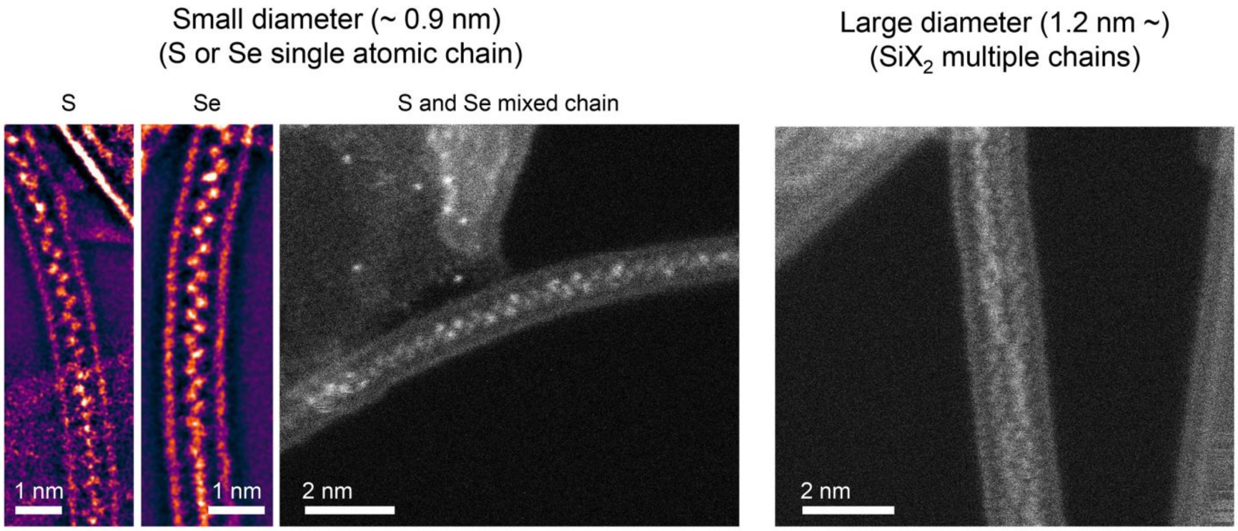

Supporting Figure S10. Extra STEM images of other structures inside nanotubes.

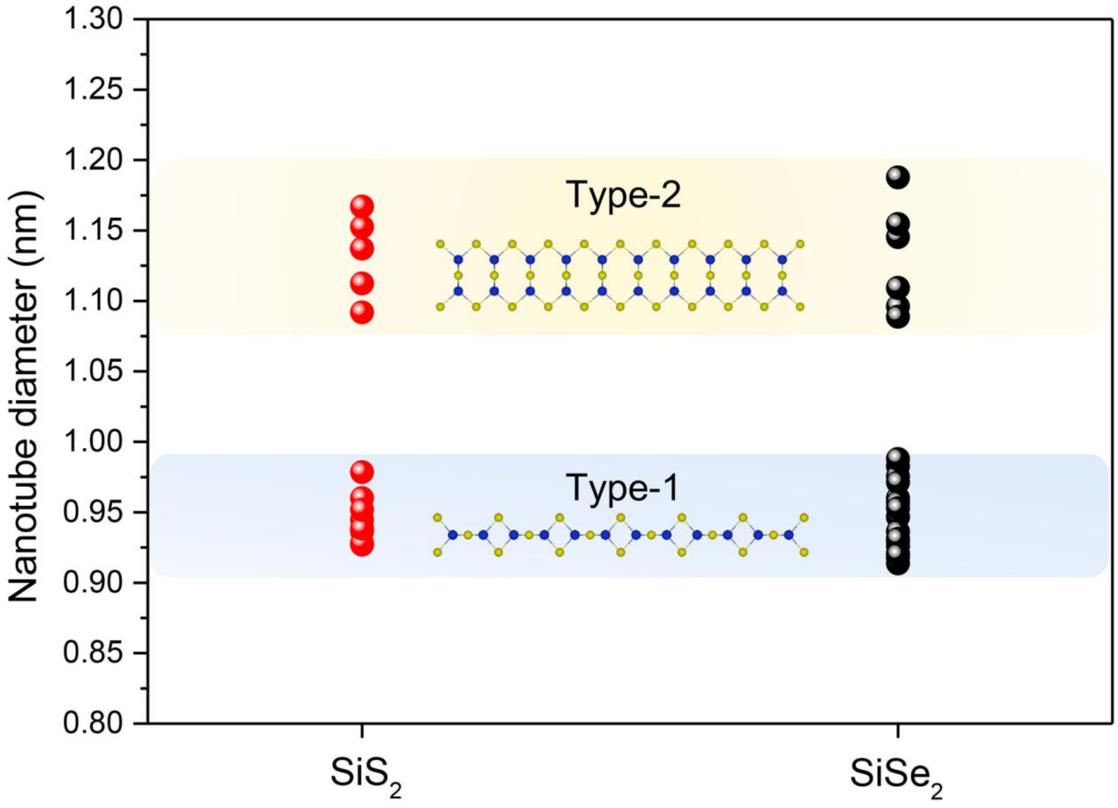

Supporting Figure S11. Nanotube inner diameter dependence.

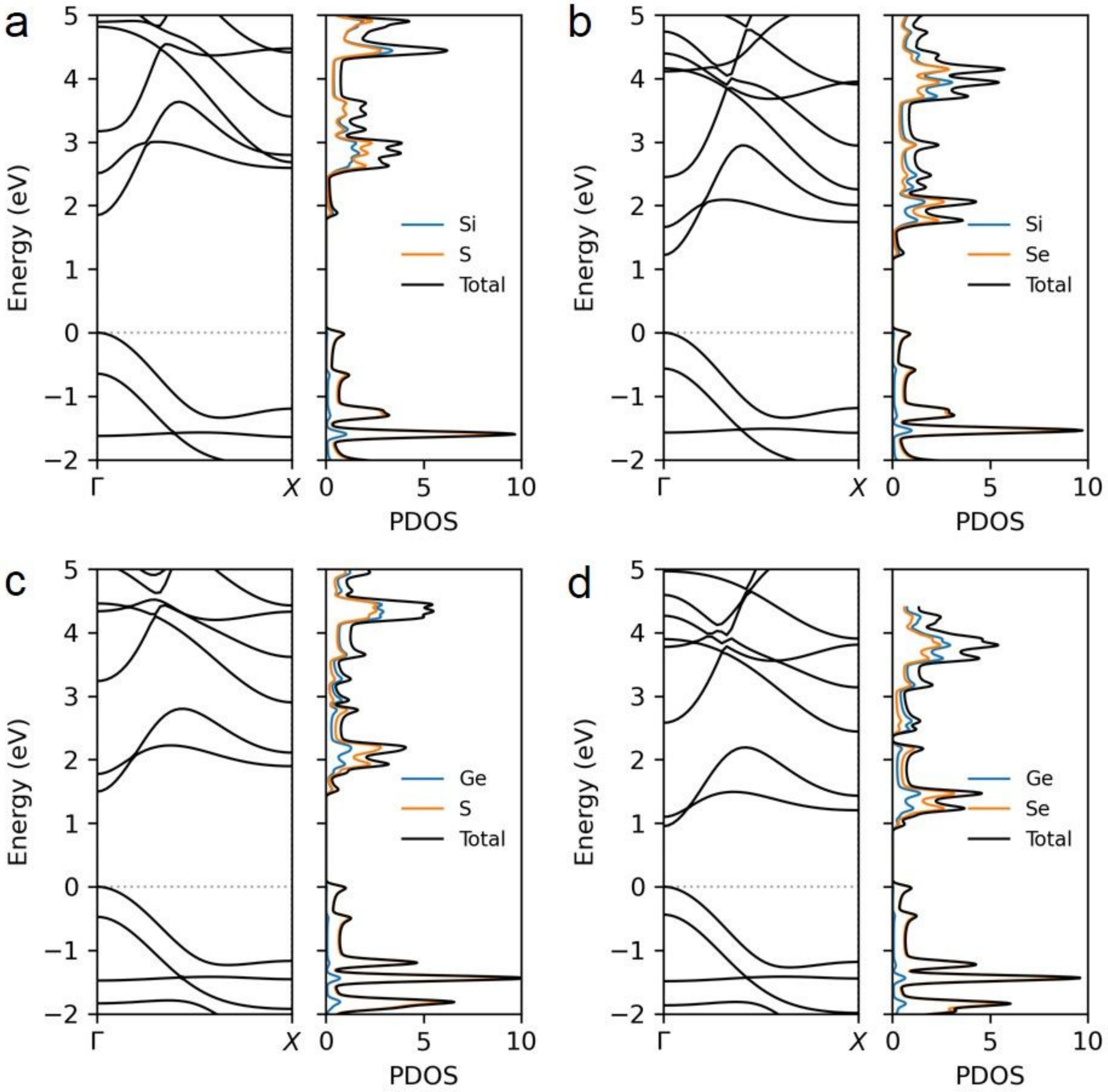

Supporting Figure S12. Calculated band structures and projected density of states (PDOS) of type-2 single-chain (a),(b) SiX_2 (X = S and Se) and (c),(d) GeX_2 for comparison.

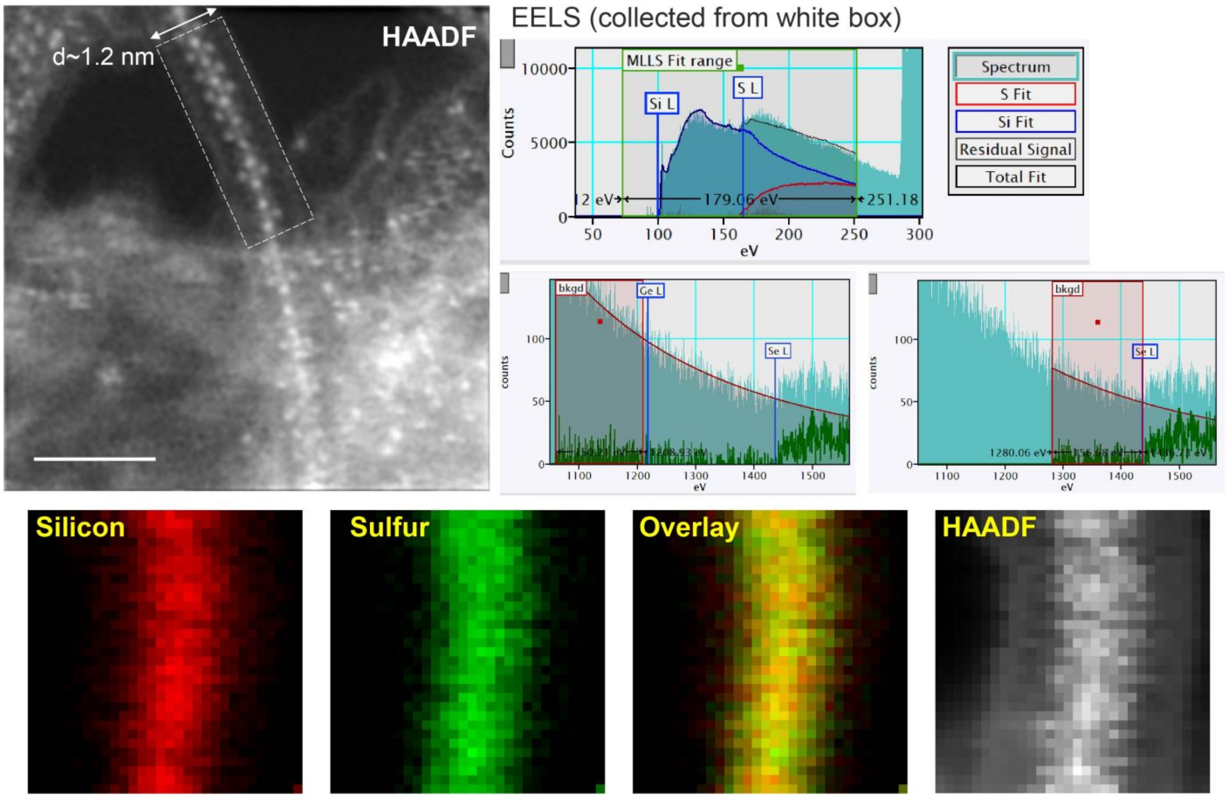

Supporting Figure S13. EELS characterization of SiGeSSe alloy chain inside nanotube. Scale bar: 2 nm.

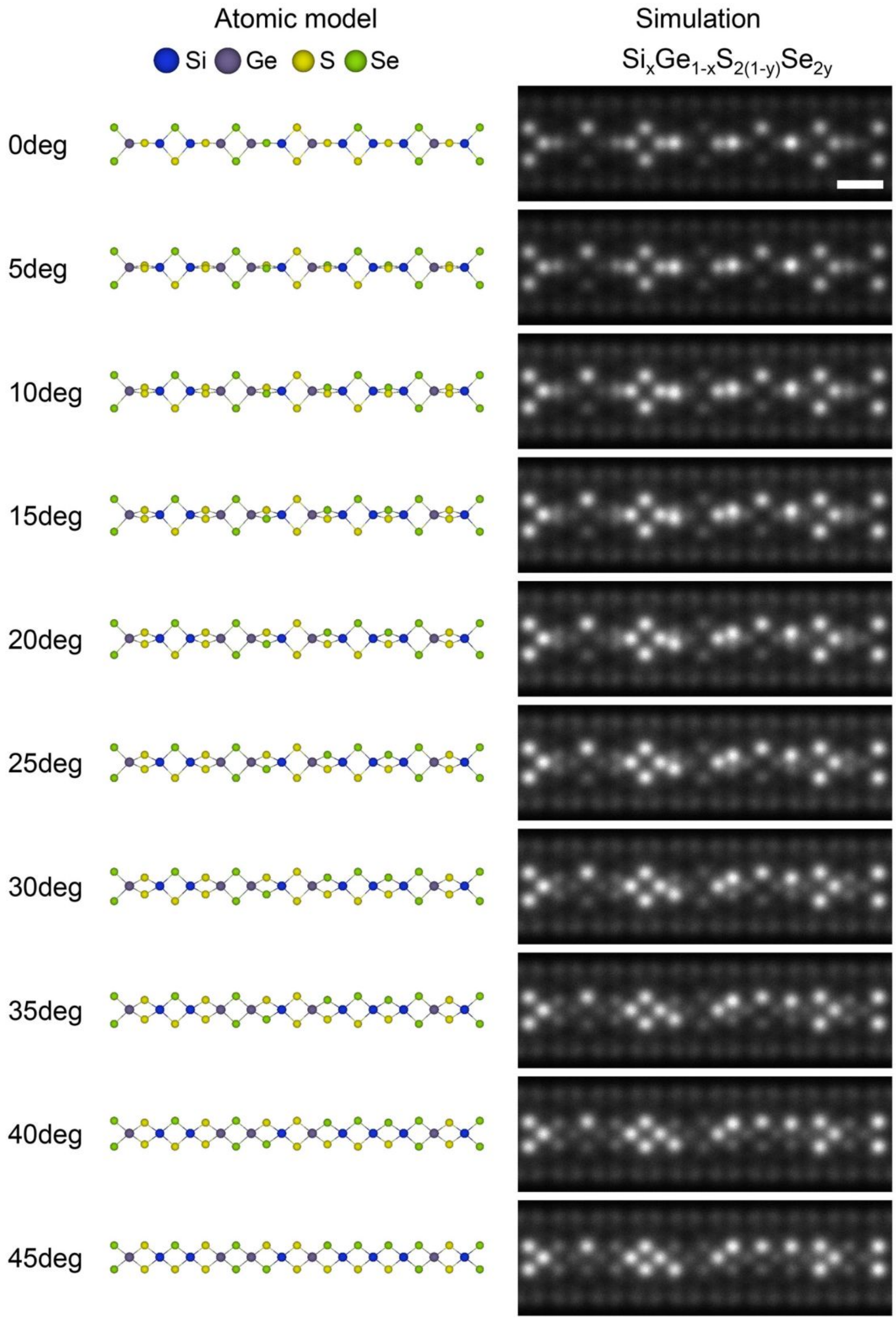

Supporting Figure S14. Simulated STEM images of type-1 $Si_xGe_{1-x}S_{2(1-y)}Se_{2y}$ alloy chain inside a nanotube. Scale bar: 0.5 nm.

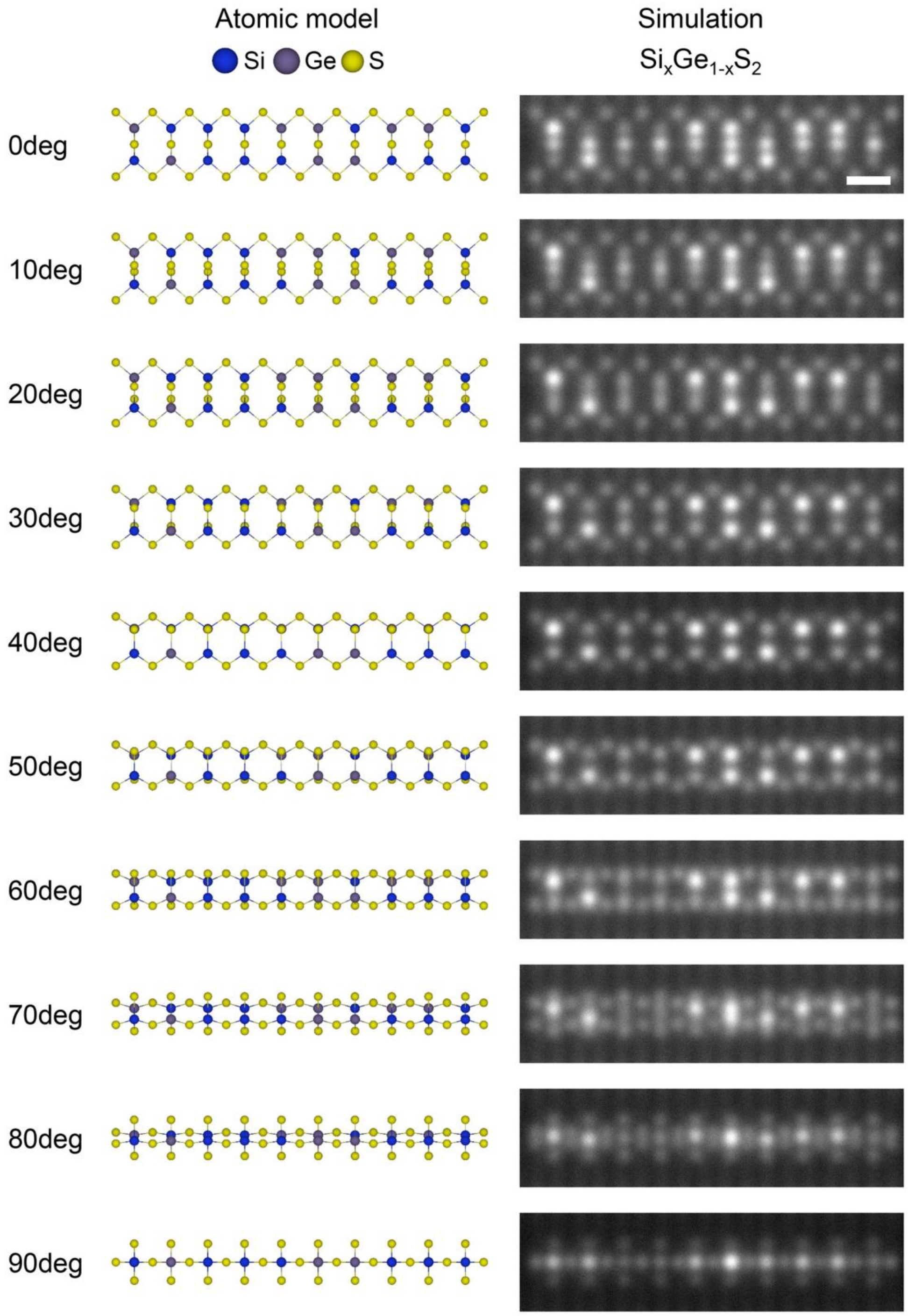

Supporting Figure S15. Simulated STEM images of type-2 $Si_xGe_{1-x}S_2$ alloy chain inside a nanotube. Scale bar: 0.5 nm.

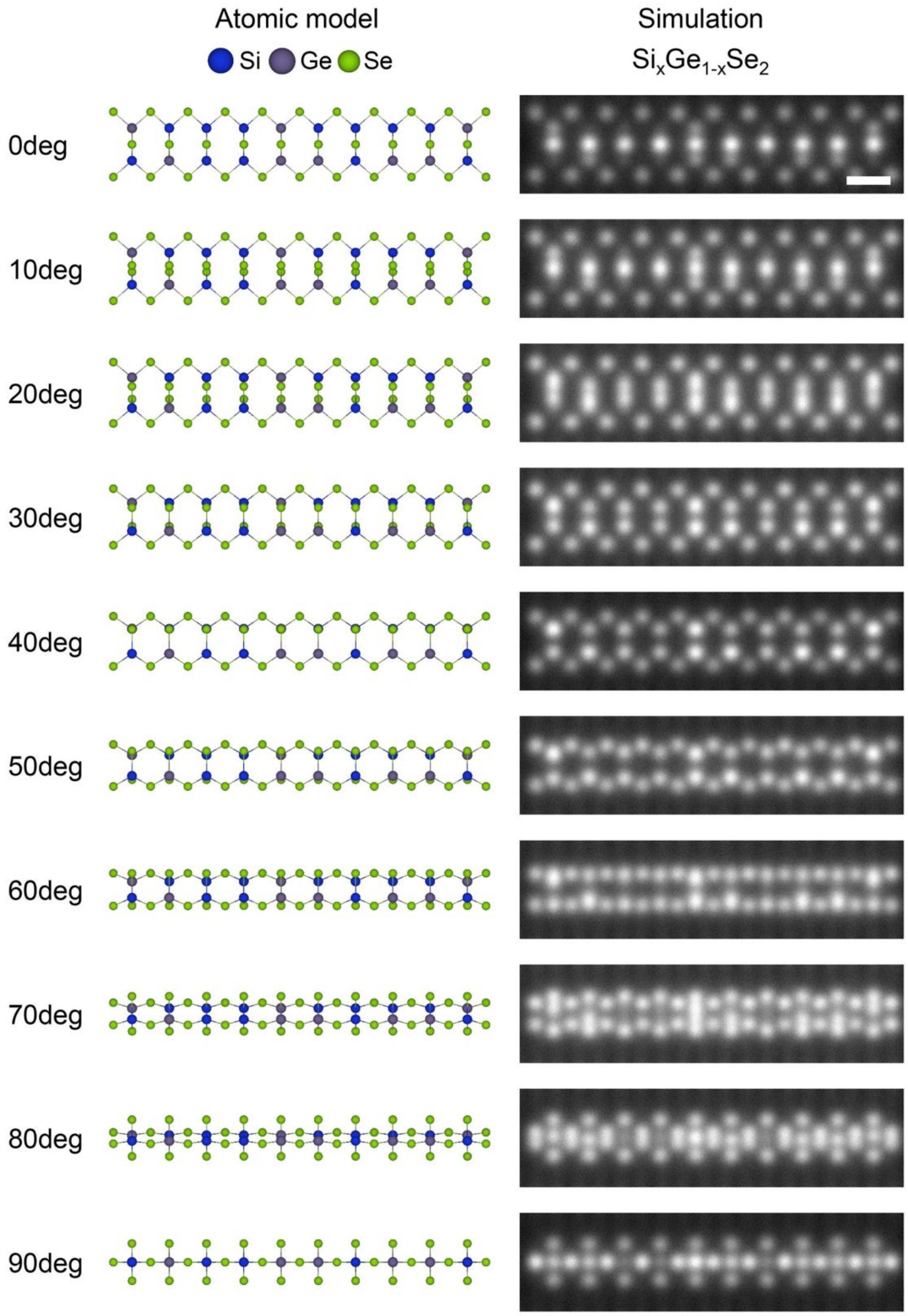

Supporting Figure S16. Simulated STEM images of type-2 $Si_xGe_{1-x}Se_2$ alloy chain inside a nanotube. Scale bar: 0.5 nm.

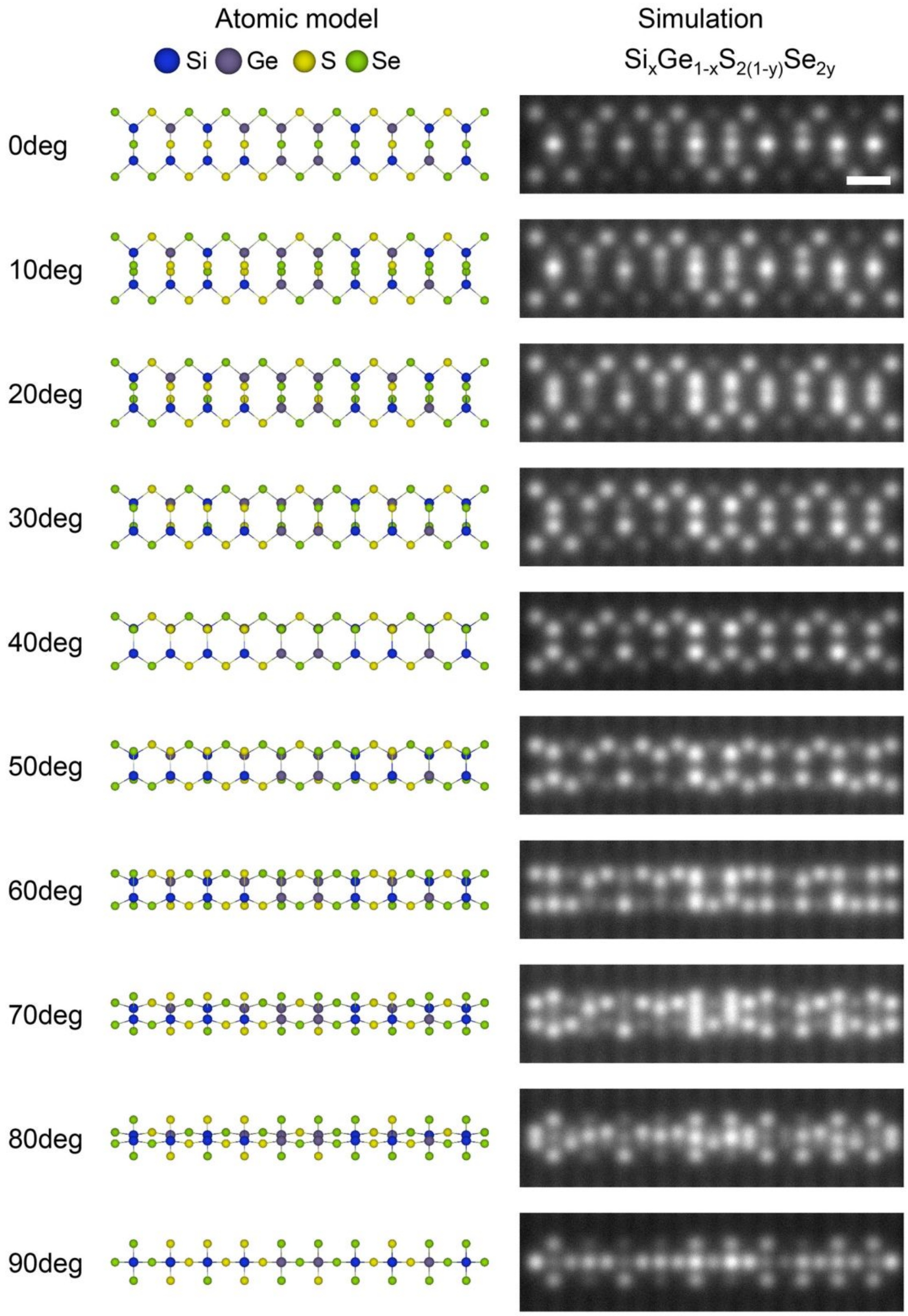

Supporting Figure S17. Simulated STEM images of type-2 $Si_xGe_{1-x}S_{2(1-y)}Se_{2y}$ alloy chain inside a nanotube. Scale bar: 0.5 nm.
